# High Speed Crop and Weed Identification in Lettuce Fields for Precision Weeding

**DOI:** 10.3390/s20020455

**Published:** 2020-01-14

**Authors:** Lydia Elstone, Kin Yau How, Samuel Brodie, Muhammad Zulfahmi Ghazali, William P. Heath, Bruce Grieve

**Affiliations:** School of Electrical and Electronic Engineering, The University of Manchester, Oxford Rd, Manchester M13 9PL, UK; hkyhow12@gmail.com (K.Y.H.); brodie667@hotmail.com (S.B.); ghazalizulfahmi@gmail.com (M.Z.G.); william.heath@manchester.ac.uk (W.P.H.); bruce.grieve@manchester.ac.uk (B.G.)

**Keywords:** precision weeding, multispectral imaging, kinetic stereo imaging, plant detection

## Abstract

Precision weeding can significantly reduce or even eliminate the use of herbicides in farming. To achieve high-precision, individual targeting of weeds, high-speed, low-cost plant identification is essential. Our system using the red, green, and near-infrared reflectance, combined with a size differentiation method, is used to identify crops and weeds in lettuce fields. Illumination is provided by LED arrays at 525, 650, and 850 nm, and images are captured in a single-shot using a modified RGB camera. A kinematic stereo method is utilised to compensate for parallax error in images and provide accurate location data of plants. The system was verified in field trials across three lettuce fields at varying growth stages from 0.5 to 10 km/h. In-field results showed weed and crop identification rates of 56% and 69%, respectively. Post-trial processing resulted in average weed and crop identifications of 81% and 88%, respectively.

## 1. Introduction

Traditionally, weed management has been achieved through broadcast application of selective herbicide. A large proportion of herbicide used in broadcast spraying is released into the environment through run-off and drift [1]. Increasing environmental and public health concerns have resulted in stricter regulation of herbicide use [2,3]. Traditional selective herbicides also have to contend with a growing number of resistant weed species [4]. Weeds represent a loss potential of 32%, with an effect comparable to that of pathogens and parasites combined [5]. Therefore, effective weed control is essential to maintaining and increasing worldwide food productivity to provide for a growing global population [6]. Speciality crops, such as vegetables, are disproportionally affected by herbicide resistance, as very few herbicides are registered for use in the sector [7]. Such farmers have been forced to use hand-weeding methods, which are expensive, inefficient, and made more difficult by an industry-wide labour shortage [8,9].

Several approaches have attempted to tackle weed management issues through the introduction of new technology. Herbicide waste can be moderately reduced through the use of variable-rate spraying and modifying existing spray booms [10]. Herbicide inputs may be completely eliminated through robotic weed removal [11,12]. These approaches can be implemented using relatively simple detection techniques, estimating overall plant coverage [10,13] or crop position [12,14]. Individual weed targeting for mechanical removal [15] or herbicidal micro-dosing [16,17] has also been investigated in recent years. Non-selective herbicides less effected by herbicide resistance can be utilised for micro-dosing systems, which can reduce herbicide requirements by up to 99.3% according to [18]. In order to achieve individual weed targeting, plant identification and accurate target position information are essential.

Weed detection methods most often use a combination of a colour index followed by image segmentation and feature extraction, to locate weeds in the crop bed [19]. Other methods have been considered, but not so extensively, such as the use of LIDAR (light detection and ranging) to discriminate between crops, plants, and soil using height information [20]. Colour indices use some combination of visible and near infra-red (NIR) reflectance to create a single greyscale image [19,21]. Some methods require the transformation into different colour spaces [22], such as normalised RGB [23] or HSV (hue, saturation, and value) [24]. Common indices include ExG (excess green), ExR (excess red), NDI (normalised difference index), and NDVI (normalised difference vegetative index), which discriminate between plants and soil with varying effectiveness [19]. Images are segmented using a variation of thresholding techniques, but most commonly Otsu’s thresholding technique [21]. To discriminate crops from weeds, other information is needed; shape [25] or textural features [26] are most commonly used, which can be computationally expensive [3].

Machine learning has been used extensively in the discrimination of vegetation and soil and between vegetation types. Support vector machines proposed in [27,28,29] have been utilised, in combination with a selection of features, such as colour or shape information, for plant identification. Fuzzy decision making methods were considered in [25] for the identification of monocot and dicot weeds, using a set of shape features achieving up to a 92.9% classification accuracy. Each of these approaches for decision making is applied following the segmentation of the image into crop and weed areas and the calculation of a selection of features for plant areas. Convolutional neural networks (CNN) have also been utilised successfully in [30,31] to locate weed patches, without the need to provide feature sets prior to processing. While the method increases the training time for the algorithm compared to, for example, support vector machines, it may provide a more universally applicable system [31]. These methods show an important next step in the identification process, but are complex and relatively computationally expensive. They are most notably being used to identify patches of weeds within a field, not individual weeds in real-time.

**Figure 1:**
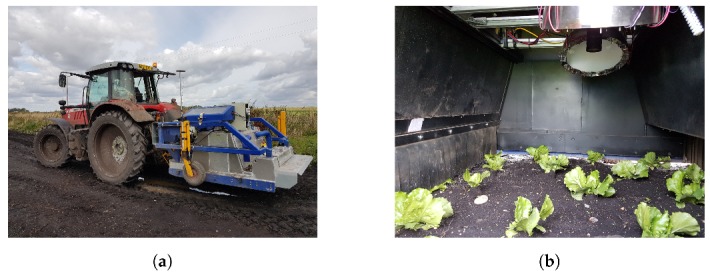
The system in field trials. (**a**) The system mounted to the rear of the tractor. (**b**) The sensor system in-field.

This study focuses on the development of a low cost, high-speed detection of individual plants for use with a herbicide micro-dosing system, assuming a targeting area 20 mm in diameter. As such, our detection system was designed with the target of operating at 5 km/h with the capacity for cost-effective retrofitting to existing tractor tool-bar mounts, and minimal equipment cost. The approach should be robust to variations in ambient lighting and to inadvertent wind dispersion on the injected herbicidal products. The assembly, therefore, is enclosed under a tractor-mounted hood (Figure 1) and controlled illumination is provided. The research aimed to determine the effectiveness of using spectral reflectance in the red, green, and NIR wavebands using controlled illumination, combined with size information in the discrimination between crops, weeds, and soil in horticultural crops. Accurate position information for individual weed targets is required whilst maintaining a large field-of-view from the camera to ensure high-speed operation. A system to mitigate the effect of parallax error on location information of plants has been proposed through a kinematic stereo method. Where possible, the detection method should provide flexibility for use across a range of scenarios. As such, a modular design was utilised to allow for customisable width up to a maximum of seven modules (3.78 m wide), which may be implemented concurrently for each PC.

## 2. Materials and Methods

### 2.1. Hardware Design

Each camera-lighting module (Figure 1b) covers a width of 0.54 m, allowing for customisation according to application. Weather, time, and location may cause significant variations in the imaging environment, and the detection system must be robust to these changes. Therefore, a closed canopy houses the imaging system to provide illumination control. For easy integration with current farming equipment, the canopy is mounted to the rear of a tractor (Figure 1a) and covers one crop bed (1.6 m, 3 camera-lighting modules).

Three main components are used for crop and weed identification: a PC, RGB camera, and lighting rig; a 12 V lead-acid battery powers the system in conjunction with an inverter to supply the PC; LED arrays, 650 (red), 850 (NIR), and 525 nm (green) (Justar Electronic Technology Co. LTD). 5 × 5 arrays at 525–530 nm, 650 nm, 850 nm, 25 W, 1.75 A are used for illumination.

**Figure 2:**
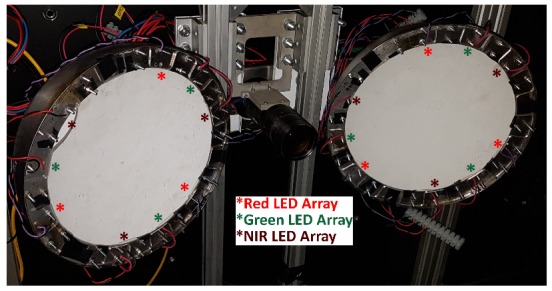
Illumination system containing nine LED arrays per lamp, positioned at regular intervals.

An RGB machine vision camera (PointGrey, Grasshopper 3, GS3-U3-41C6C-C), with a wide angle lens (Fujinon CF12.5HA-1), is used for image capturing. The camera is mounted between two lamps to provide shadowless illumination. Each lamp consists of three LED arrays of each wavelength placed at 120° intervals within a metal hemisphere (Figure 2). The camera is positioned 0.6 m above the ground resulting in a field of view of 0.54 × 0.54 m and a resolution of 0.9481 px/mm. This height was chosen to balance coverage and precision requirements, whilst minimising image distortion.

Illumination control is facilitated through two circuit boards: the LED driver and microcontroller boards. The camera receives signals from the PC to capture images, which are relayed to the microcontroller board. The microcontroller generates PWM signals to control the intensity of the LED arrays of different wavelengths, and multiplexes these with the ON/OFF signals received from the camera. The LED drivers take this control signal and a 12 V supply to illuminate the area when required by the camera. The duty cycles for each of the LED arrays are 50%, 50%, and 20% for the NIR, green, and red, respectively. Comparisons were made between a selection of images taken at varying duty cycles (between approximately 10% and 70% for each wavelength). Where the duty cycle for each wavelength was equal, the red response appeared to dominate the image. The combination of duty cycles chosen allowed for a balanced response in each channel and resulted in an image with clearly differing responses between plants and soil, and to a lesser extent between plant types.

A custom built PC provides the required processing power for high speed identification, including a dedicated GPU. The full PC component list can be seen in Table 1.

### 2.2. Plant Identification

Our approach uses red, green, and NIR reflectivity, combined with an assumption that crops are generally larger than weeds, to identify crops and weeds in-field. In [32] a modified RGB camera is used to implement NDVI using multiple filters to allow the green and blue channels to detect NIR wavelengths. Our approach similarly modifies an RGB camera through the removal of the NIR filter and introduction of a 515 nm long-pass filter to remove the unwanted response to blue light in the blue channel. However, in addition to utilising red and NIR wavelengths, the green response is maintained from the camera. The green response is intended to obtain minor tonal differences between plant types to be included in the index calculation (RGNIR—Equation (1)). This may be useful where leaves of crops and weeds overlap or large bodies of multiple weeds appear to be a single larger object.

A single shot method is used, flashing all LEDs simultaneously and capturing the red, green and NIR reflectance in one frame. The RGB channels of the camera overlap, and as such, all respond to the wavelengths used in the system to a varying extent. From the datasheet for the CMOSIS CMV400 chip, an estimation of the quantum efficiency of the RGB channels at the system wavelengths is shown in Table 2.

**Figure 3:**
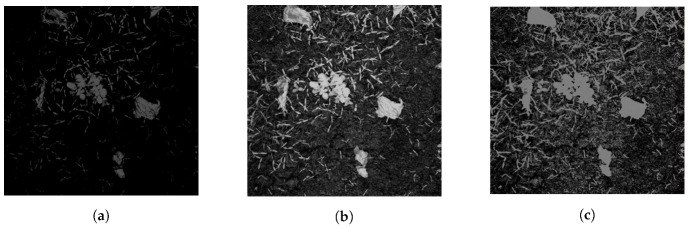
Responses of the red (**a**), green (**b**), and blue (**c**) camera channels, in controlled illumination.

The removal of the NIR filter led to colour distortion of the captured image; to compensate, a background image is taken in a black box condition and then removed from the image. An example of each of the captured RGB frames is shown in Figure 3. The channel responses are combined in Equation (1) to produce a single greyscale image for segmentation. This equation is similar to the normalised green index proposed by [33], although in our case the blue channel is a combined response to NIR and green wavelengths. The RGNIR intensity is calculated as

(1) $$RGNIR=\frac{\beta (G_{c}-g_{c})}{\alpha \left(R_{c}+r_{c}\right)+\beta \left(G_{c}-g_{c}\right)+\gamma \left(B_{c}-b_{c}\right)+L},$$

where $G_{c}$, $R_{c}$, and $B_{c}$ are the green, red, and blue channels, respectively; $g_{c}$, $r_{c}$, and $b_{c}$ are the background corrections for each channels (0–255); *α*, *β*, and γ are the channel weights (0–1); *L* is the soil adjusted constant.

**Figure 4:**
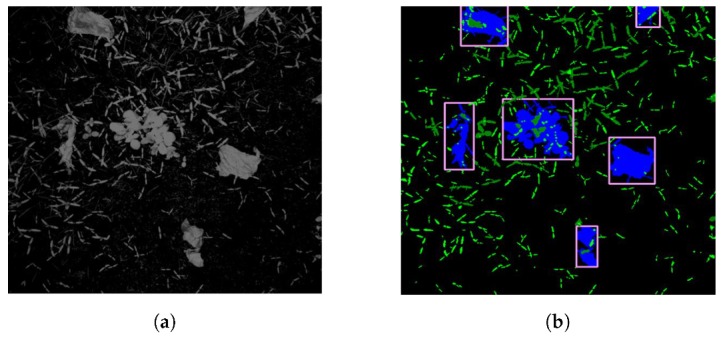
Image processing steps. (**a**) Greyscale output of the RGNIR function. (**b**) Image following thresholding.

The histogram of the RGNIR image (Figure 4a) is taken and segmentation is performed using Otsu’s multi-level (in this case three) thresholding method [34,35]. The features from the segmented image are identified as either crop or weeds using a size exclusion method (Figure 4b).

### 2.3. Height Estimation

**Figure 5:**
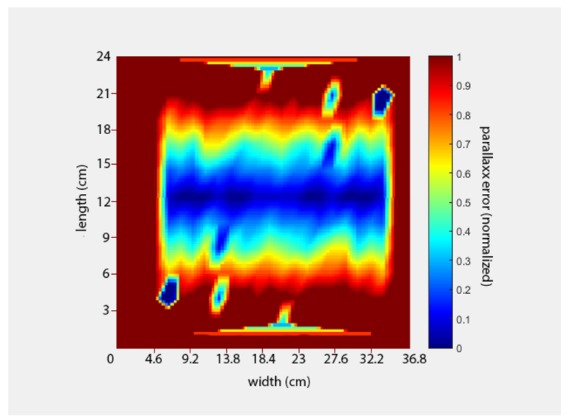
Heat map of the normalised position error across a frame.

In order to image a field efficiently at high speed, it is essential that each frame captures the largest feasible area while maintaining sufficient detail to accurately identify and locate small plants. However, where plants are not located directly below the camera, variations in their height can result in an inaccurate determination of their position due to parallax error. In our system, the usable frame area (assuming a parallax error of less than 5% is acceptable) is approximately a 19% slice across the centre (Figure 5).

While it is possible to disregard all but this area within the image, the camera would need to capture five times more images in order to cover the same area. This effectively reduces the camera frame rate from 90 fps to ≈17 fps, limiting the maximum speed of the system and the ability to incorporate additional wavelengths in the future. A stereo imaging technique using a single camera in motion [36,37] was chosen to compensate for the error, eliminating the need to purchase additional equipment.

Given that the imaging system is in constant motion, and the camera has a high frame rate, it is possible to use a single camera to achieve kinetic depth estimation. As the camera moves between two consecutive images, taller objects will appear to move faster than their shorter counterparts. Therefore the height disparity between the objects can be calculated using a dense optical flow algorithm. The system utilises the Farneback optical flow algorithm [38], chosen because of the low processing time and availability of the GPU kernel in the OpenCV library.

**Figure 6:**
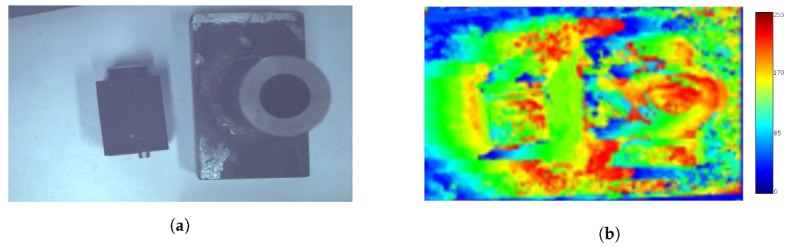
Height estimation using kinematic stereo method—preliminary test. (**a**) The image prior to processing containing two objects of different heights. (**b**) The height disparity map produced using the optical flow algorithm on the first attempt.

Tests were carried out to verify the reliability of the algorithm using objects of known height imaged twice with 2.1 cm camera displacement between images (chosen from the 90 fps camera frame rate and target 5 km/h system speed). The item on the left in Figure 6a is 2.5 cm tall; the object on the right is 3.5 cm tall at its lowest point and 11 cm at its highest. A height disparity map was produced from these images (Figure 6b), which shows significant error in the height estimation algorithm. The error was attributed to the small number of features, and thus, low spatial frequency of the image. Given that neighbouring pixels had very similar values, the algorithm was not able to accurately calculate the pixel shift.

**Figure 7:**
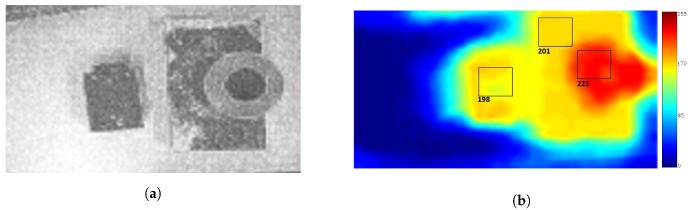
Height estimation using the kinematic stereo method with the addition of a Laplacian filtered image. (**a**) Sum of the original and Laplacian filtered images. (**b**) The height disparity map using the optical flow algorithm with addition of laplacian filtered image to reduce error.

The error in the disparity values can be reduced by applying a Laplacian filter to the image and summing the filtered and non-filtered images (Figure 7). This can improve the texture of the image, preserving the high spacial frequency components. As can be seen in Figure 7, the error in disparity values across the image is reduced. Approximately 1 cm in height corresponds to 3 units in the height disparity map (Figure 7), showing a good estimation of the object height by the algorithm. However, some inconsistencies still exist at object edges, which can be mitigated by taking the disparity value in the middle of any given object.

Using Equation (2) the real distance can be calculated, allowing the system to provide accurate position information of individual plants across an entire frame. The distance between two frames is calculated using a speed estimation algorithm and the time between images, as:

(2) $$x-x^{\prime }=\frac{bf}{d}$$

where *x* and $x^{\prime }$ are the point of interest in frame one and the one in frame two respectively; *b* is the distance between frames; *f* is the focal length of the camera; and *d* is the disparity map (object height).

### 2.4. System Optimisation

The system uses a NVIDIA GTX1080 GPU with a parallel implementation of the software developed in NVIDIA Compute Unified Device Architecture (CUDA)—Toolkit 9.0. A parallel prefix sum algorithm is used to reduce the run time of the multi-thresholding algorithm by several orders of magnitude. Three images were processed 1000 times, and the average speed was taken for the implementations on each the CPU and GPU to establish the time advantage of introducing multithreading. The results show the algorithm is up to 10,000 times faster using the GPU method, with the same threshold values found. A trial run of 50 images gave a total average loop time of 100 ms and maximum of 175 ms per frame with one detection module in use.

**Figure 8:**
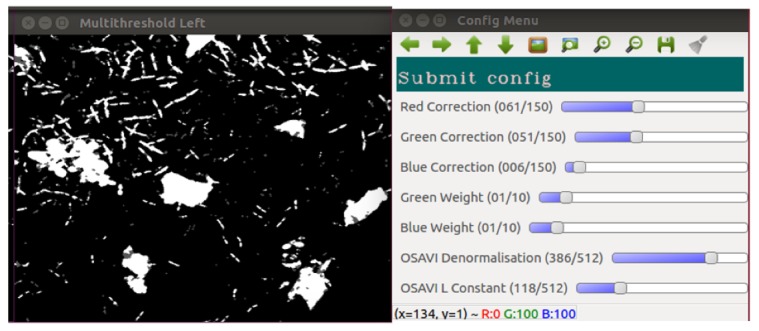
Screenshot of the system during calibration process for plant identification, showing a desirable output following thresholding.

An in-field calibration method allows the user to optimise the system for different conditions. A background image is taken with no illumination, as are two further images in different positions to allow the user to tune parameters in Equation (1) and the height estimation algorithm. Figure 8 shows the calibration process with the “ideal” processed images output from the tuned parameters.

### 2.5. Field Trial Procedure

Trials were performed in three separate iceberg lettuce fields at three growth stages in Ely, Cambridgeshire, United Kingdom on the 17 August 2018.

The tractor speed was varied from 0.5 to 10 km/h. The average weed density varied across each of the fields (150, 69, 31 weeds/m^2^ for fields 1, 2 and 3 respectively). A maximum density of 364 weeds/m^2^ was found in those frames analysed. Weeds found in the fields were between the one and three leaf stage and were predominantly broadleaf type.

Over 20,000 unprocessed images were captured from the trials for further testing. Each image file name contains the timestamp of capture, such that any lag in the system may be detected. The time between frame capture is fixed regardless of system speed, resulting in increased overlap between frames at a lower speed. This variable could be optimised to reduce overlap at lower speeds for future trials. Each trial run produced a database entry for each crop detected, including its size and location. These databases were produced to log information which may be of use to the farmer in other activities.

**Figure 9:**
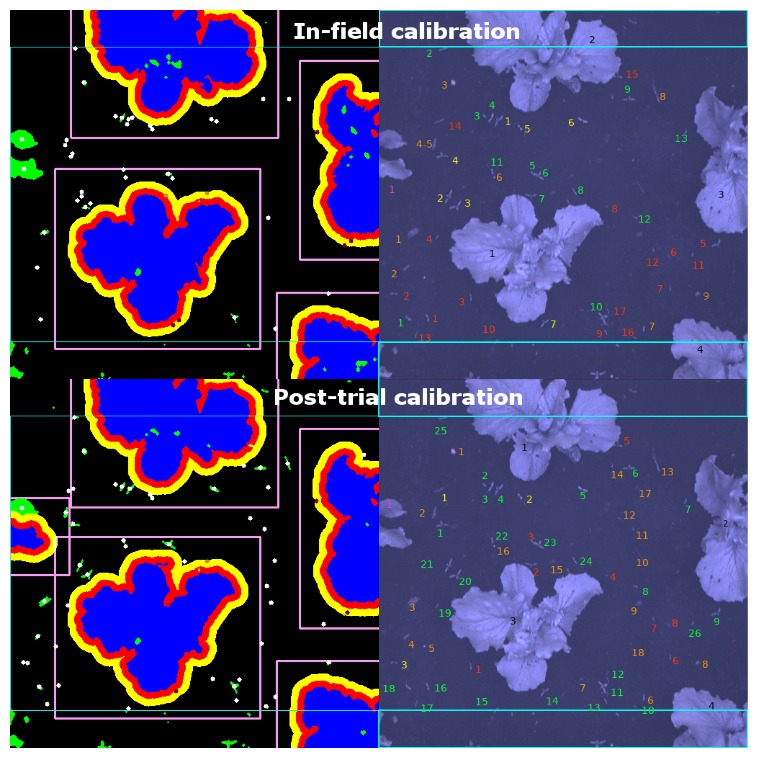
Images following processing (**left**) using in-field (**top**) and post-trial (**bottom**) calibration. Blue areas were designated as crops, and weeds are shown in green, with the centre of each target identified by a star. Unprocessed images are labelled by hand (**right**) according to the accuracy of the system identification using the key as defined in Table 3.

The in-field calibration files were also saved to reproduce the processed images after the trial; there were two files, one used for field 1 and part of field 2, the other for the remainder of field 2 and field 3. A random sample of 50 images was considered for further analysis. Each of the raw images was evaluated, and weeds and crops were identified by eye. This was then compared to the processed images to identify which plants had been correctly identified and where false negatives and positives had occurred according to the colour code described in Table 3. The process was completed first with the trial calibration and then with the post-trial calibration to establish the limitations of the calibration process and the capabilities of the system with optimal calibration (Figure 9).

Crops are encircled with yellow and red regions to indicate the area within 2 and 4 cm of a crop respectively. Targets within the red-zone are ignored, as they are considered likely to result in crop damage, those within the yellow-zone are flagged to be handled with care, as are those within the pink box. Blue boxes at the top and bottom of each image identify the edge of the area for targets to be generated within each frame. The post-trial set used also used two calibrations, one for field 1 and another for field 2 and 3; this is mainly due to the significant size difference between the crops in these fields.

## 3. Results and Discussion

**Figure 10:**
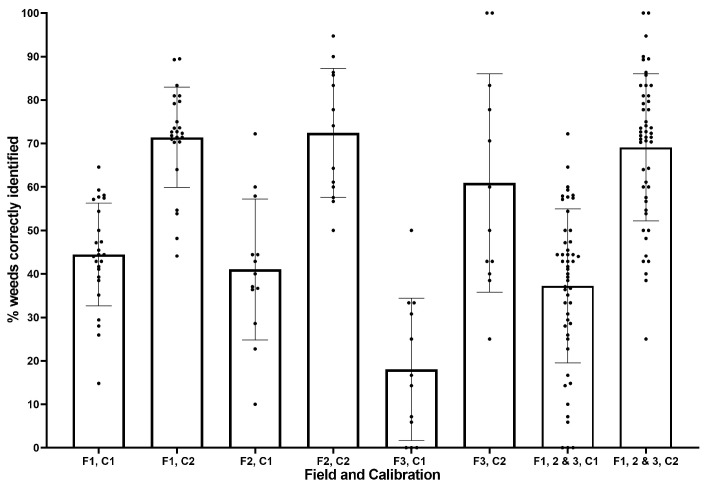
Proportion of weeds correctly identified for each field (F1–3) and calibration (C1, C2).

Figure 10 shows the proportion of weeds per frame correctly identified taken from a sample of 50 of the processed images. Post-trial calibration (C2) results are consistently better than their in-field (C1) counterparts. With increasing field number, positive weed identification decreased for both in-field and post-trial calibration, with a particularly poor result (18%) for field 3 in-field calibration.

Field 3 had the smallest crops, and therefore, the fewest weeds/frame on average (nine) which may have resulted in the smaller peak in the histogram, a change in the threshold values, and a worse detection rate. Field 3 and part of field 2 also used a second, different calibration in-field due to the substantial change in crop size from those in field 1. This in-field calibration may have been less effective than that used for field 1. Post-trial calibration results are an improvement due to a process of trial and error not possible in-field and due to a lack of screen visibility in-field making it difficult to finely tune as required.

**Figure 11:**
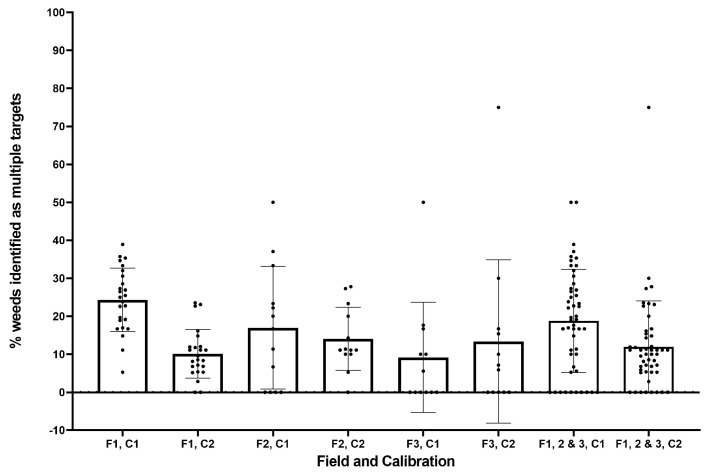
Proportion of weeds identified as multiple targets for each field (F1–3) and calibration (C1, C2).

**Figure 12:**
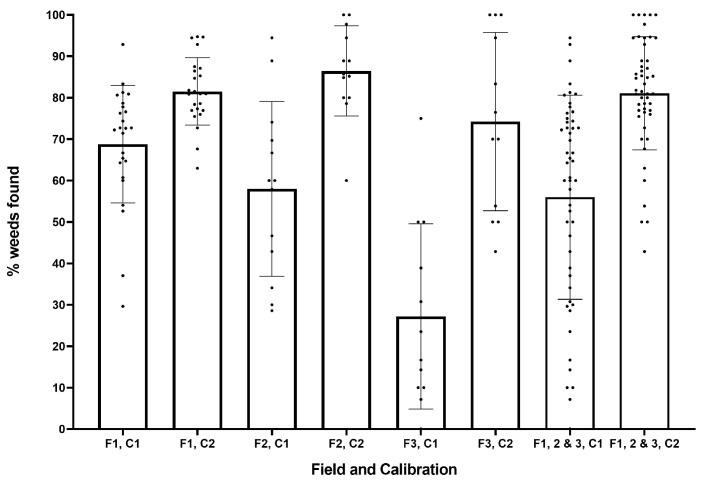
Proportion of weeds identified for each field (F1–3) and calibration (C1, C2).

As can be seen from Figure 11, there was a wide range of values for the percentage of weeds identified as multiple targets, particularly in field 3. For increasing field number, there was a decreasing trend of multiple target weeds. Larger weeds with several leaves are most often misidentified as multiple targets, where each leaf is mistakenly identified as an individual plant. Those fields with fewer and smaller weeds are less likely to have weeds identified as multiple targets. Overall, the post-trial calibration reduces this error in identification (from an average 19% to 12%). Post-trial results in field 3 do not appear to reduce the error as expected; however, the wide range in results makes it difficult to draw a definitive conclusion from these results. Although the addition of extra targets to the system may decrease efficiency, these weeds can still be effectively managed, and the overall effect is determined by the resolution of any actuator system. As such, it is clear the system performs well in identifying weeds, with total average identification rates of 56% and 81% for the in-field and post-trial calibrations respectively (Figure 12).

**Figure 13:**
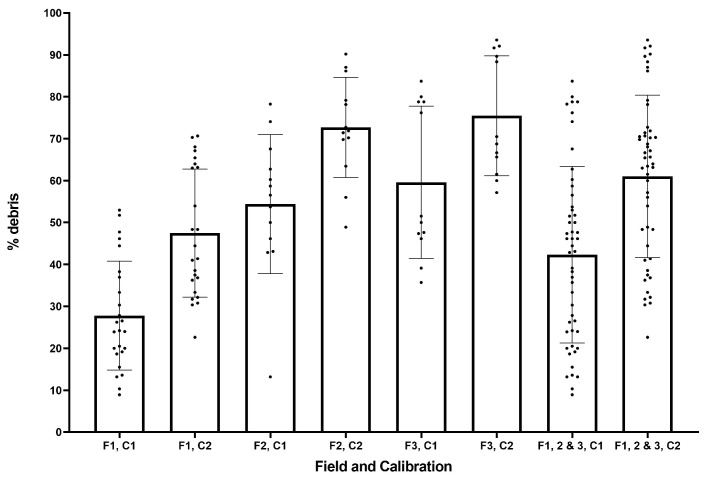
Proportion of objects identified which are debris for each field (F1–3) and calibration (C1, C2).

The system has a significant problem with false positives, where debris in the field (e.g., stones and twigs) are incorrectly identified as a weeds. Whereas weed identification improves in the post-trial calibrations, false positives worsen, and there is a clear trade-off when calibrating the system between improved weed identification and increasing false positives. Figure 13 shows the proportion of generated targets which are in fact debris.

Field 1 had the lowest proportion of misidentified targets, due to its higher number of weeds/frame (average of 43 weeds/frame). The larger number of weeds, if average debris per image is approximately constant, results in an apparent reduction of debris identified. Where there is higher leaf coverage in an image, more of the debris will also be obscured. Finally, where there is a very high debris-to-weed ratio, it may result in a false peak in the histogram and error in identification.

**Figure 14:**
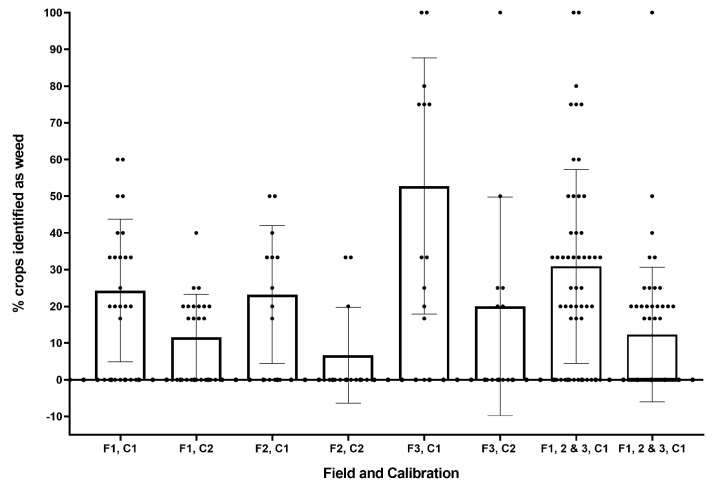
Percentage of crops misidentified for each field (F1-3) and calibration (C1, C2).

Approximately 31% and 12% of crops were misidentified as weeds in the in-field and post-trial calibrations respectively, as shown in Figure 14. The vast majority of these false positives were at the edge of images where the crop is not fully visible, and as such the size exclusion method identified the object as a weed (see Figure 9). The effect was most pronounced in field 3, where crops were smallest. This issue could be mitigated in future work using image stitching when more than one module is in use so the full crop is visible to the system. Alternatively, as in [39], plants not fully contained in the image may be excluded from the classification algorithm to avoid crop damage. The increase in crop misidentification in in-field calibration is due to the threshold for size exclusion being set too high. The size exclusion threshold is currently taken from approximate crop size provided during calibration. It may be more effective to derive the value instead from weed size, as there is considerable variation in crop size within each field.

**Figure 15:**
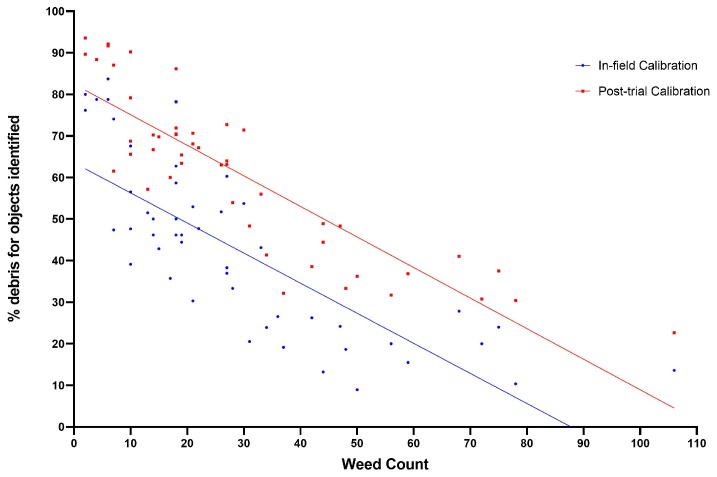
Proportion of objects identified which are debris versus weed count.

**Figure 16:**
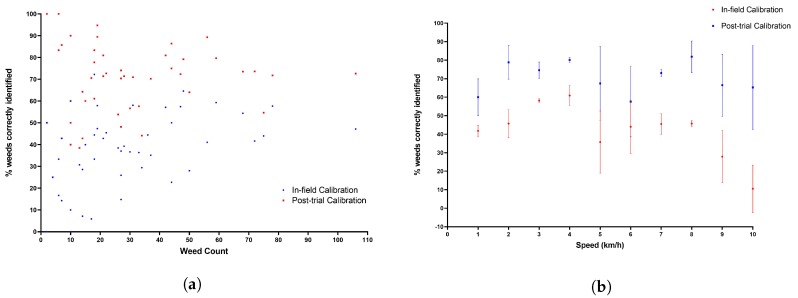
System weed identification capability with varying speed and weed count. (**a**) Percentage of correctly identified weeds versus total weed count. (**b**) Percentage of correctly identified weeds versus system speed in-field.

Figure 15 and Figure 16a show the debris misidentification and percentage of weeds correctly identified respectively, with respect to weed count. No clear correlation is shown between weed count and correct weed identification or speed and weed identification (Figure 16b). It should be noted that at the highest speeds results may deteriorate as images become more blurry, and identification by eye of objects in raw images becomes more difficult. However percentage of debris and weed count are clearly correlated for the reasons discussed previously.

It should be noted that some lag in the capture of a very small minority of images occurred during the field trial; the reason for this is unclear and should be considered in future studies. Due to the significant overlap of each frame in most of the trial speeds, this did not result in the failure to identify objects in the areas were this error occurred. Individual weed and debris identification may have resulted in the introduction of some human error to the determination of the effectiveness of the system.

These results are promising but further development should be considered to increase classification rates and reduce false positives. The system provides a relatively simple approach for crop and weed identification, but more complex approaches have shown better classification performance. In [28], plants were divided first into three groups of monocot, dicot, and barley with 97.7% accuracy, and weeds further classified by species with varying success using a support vector machine and shape features. Hyperspectral imaging is suggested for species identification in [40], providing 100% crop recognition, and weed species identification (31%–98% correctly identified depending on species and classifier method). This method is implemented at low resolution and operating speeds (average ground speed = 0.09 m/s). Plants were identified in maize fields using a selection of nine colour indices combined with support vector data description in [41] achieving up to 90.79% classification, but with significant variation in results due to weather and time of day. By comparison, our system has a lower classification rate, but provides real-time identification at high resolution for individual plant treatment, whilst correcting target position data through the height estimation algorithm. The system currently has sufficient computational redundancy such that future work may introduce further discriminating features and processes to improve plant recognition and reduce false positives. The need for this increased complexity to improve recognition rates may be required if it offers an economic advantage to total weed management beyond re-running the system periodically and capturing those undetected weeds from the earlier passes.

## 4. Conclusions

This paper reports a full-scale, on-tractor, field demonstration of a single-shot, multispectral imaging system for autonomous identification of emergent weeds at forward velocities of up to 10 km/h (2.8 m/s). The method successfully identified an average of 81% of weeds and 88% of crops in field trials, showing promise for the future development of this approach. The approach combines controlled spectrum artificial illumination alongside a short-wavelength (nominal cut-off wavelength, 515 nm), optically filtered, and modified (removal of NIR filter) Bayer-array (colour) digital imaging sensor. Single shot imaging of the red, green, and NIR reflectivity is delivered to achieve plant-soil discrimination.

Weeds are assumed to have a leaf area significantly less than the lettuce crop, and so a size-exclusion approach has been used to separate the target weeds from the neighbouring crop plants. This method of processing relies on the nature of lettuce production and similar horticultural crops where soil pre-treatment is used prior to the growing season to remove emergent weeds, before the transplantation of young plants. The green wavelengths (centred around 525 nm), are intended to handle more difficult weed detection cases associated with overlapping small weeds, which are then interpreted as a larger single plant. Further system testing to establish the performance at high weed density is required to verify the effectiveness of this approach.

A kinematic stereo method has been used to estimate the height of plants in the image and correct the parallax error to allow for accurate targeting of plants by a herbicide micro-dosing system. The method allows the full frame to be processed without increasing the system cost through the addition of expensive equipment, such as stereo vision cameras or LIDAR sensors. As a follow-on from this research programme, tests to ensure the proper functionality of the height-estimation system, and thereby the accuracy of the plant location data in-field are required.

Future developments of the design may include more sophisticated image processing, and taking advantage of the tonal differences between the crop and weed types or the morphologies of the various plant shapes. The significant improvement in plant identification using the post-trial calibration indicates the calibration system should be improved to ensure the system can be operated more effectively in-field.

## Figures and Tables

**Table 1:** PC components.

Component	Name
Motherboard	MSI Z370-A PRO ATX LGA1151
CPU	Intel Core i7-8700
GPU	Gigabyte GeForce^®^ GTX 1080
RAM	G.Skill Ripjaws 4 Series 16 GB
Storage	Samsung PM961
Power Supply	EVGA 600B

**Table 2:** Estimated quantum efficiency of camera channels.

RGB Channel	Wavelength		
	Red (650 nm)	NIR (850 nm)	Green (525 nm)
**Red Channel**	45%	15%	5%
**Green Channel**	3%	15%	45%
**Blue Channel**	5%	15%	15%

**Table 3:** Key for labelling of system targets.

Actual Object	Identified By System As:				
	Weed	Crop	Debris	Missed	Multiple Targets
**Weed**	Green	Blue	Orange	Red	Yellow
**Crop**	Pink	Black	N/A	N/A	N/A
